# Genomic analysis as a tool to infer disparate phylogenetic origins of dysembryoplastic neuroepithelial tumors and their satellite lesions

**DOI:** 10.1038/s41598-022-26636-7

**Published:** 2023-01-13

**Authors:** Yeajina Lee, Jeyul Yang, Seung Ah Choi, Seung‐Ki Kim, Sung-Hye Park, Hyun Joo Park, Jong-Il Kim, Ji Hoon Phi

**Affiliations:** 1 Department of Biomedical Sciences, Seoul National University Graduate School, Seoul, Republic of Korea; 2 Genomic Medicine Institute, Medical Research Center, Seoul National University, Seoul, Republic of Korea; 3 Neuro-Oncology Clinic, National Cancer Center, Goyang, Republic of Korea; 4 Division of Pediatric Neurosurgery, Seoul National University Children’s Hospital, Seoul National University College of Medicine, Seoul, Republic of Korea; 5 Department of Neurosurgery, Seoul National University College of Medicine, Seoul, Republic of Korea; 6 Neuroscience Research Institute, Medical Research Center, Seoul National University College of Medicine, Seoul, Republic of Korea; 7 Department of Pathology, Seoul National University College of Medicine, Seoul, Republic of Korea; 8 Department of Biochemistry and Molecular Biology, Seoul National University College of Medicine, Seoul, Republic of Korea

**Keywords:** Cancer, Genetics, Molecular biology

## Abstract

Dysembryoplastic neuroepithelial tumor (DNET) is a low-grade brain tumor commonly associated with drug-resistant epilepsy. About half of DNETs are accompanied by tiny nodular lesions separated from the main mass. The existence of these satellite lesions (SLs) has shown a strong association with tumor recurrence, suggesting that they are true tumors. However, it is not known whether SLs represent multiple foci of progenitor tumor cell extension and migration or a multifocal development of the main DNET. This study was designed to elucidate the histopathology and pathogenesis of SLs in DNETs. Separate biopsies from the main masses and SLs with DNET were analyzed. We performed comparative lesion sequencing and phylogenetic analysis. FGFR1 K656E and K655I mutations or duplication of the tyrosine kinase domain was found in all 3 DNET patients and the main masses and their SLs shared the same FGFR1 alterations. The phylogenic analysis revealed that the SLs developed independently from their main masses. It is possible that the main mass and its SLs were separated at an early stage in oncogenesis with shared FGFR1 alterations, and then they further expanded in different places. SLs of DNET are true tumors sharing pathogenic mutations with the main masses. It is plausible that multifocal tumor development takes place in the dysplastic cortex containing cells with a pathogenic genetic alteration.

## Introduction

Pediatric low-grade gliomas (LGGs) have distinct characteristics and prognoses. Many children with LGGs have a high propensity to develop chronic intractable epilepsy without tumor progression for a long time. So-called long-term epilepsy-associated tumors (LEATs) refer to a group of LGGs with such clinical characteristics, although the terminology is not officially incorporated in the World Health Organization (WHO) classification of brain tumors^1,2^. LEATs are a diverse group of tumors, among which dysembryoplastic neuroepithelial tumor (DNET) is the prototype disease from both clinical and pathological viewpoints^3^.

DNET was first introduced in the 1980s as a novel disease of glioneuronal tumors^4^. Patients with DNET develop seizures in early childhood and adolescence that frequently lead to drug-resistant epilepsy^5^. The mechanism of seizure development in brain tumors is still obscure, but the complex, extraordinary architecture of DNETs may explain the strong epileptogenic nature of the tumor. Interestingly, many DNETs consist of composites of a tumor and a focal cortical dysplasia which is one of the most common cortical malformations. A metabolic imaging study showed that methionine uptake levels of DNETs lie between those of focal cortical dysplasia and ganglioglioma^6^. Nevertheless, DNET is a *bona fide* tumor with proliferative activities. Once considered a static and benign lesion for which only partial removal would suffice for tumor control, recent studies on DNETs have noted rather high rates of recurrence^7,8^.

Some DNETs have discrete mass-like features with well-demarcated margins on magnetic resonance imaging (MRI), whereas others have a bubbly, popcorn-like appearance with indistinct tumor borders. Approximately 50% of DNETs show one or more tiny separate nodular lesions in the subcortical white matter^9^. These satellite lesions (SLs) are small tumor nodules grossly separated from the main mass by thin layers of white matter. Our previous study demonstrated that SLs are correlated with an increased rate of tumor recurrence if they are not completely removed^9^. The pathogenesis of this peculiar architecture of SLs in DNETs is of interest, but it is unclear whether SLs represent multifocal disease or infiltration of the main tumor mass into the white matter. It is also intriguing that SLs are highly associated with persistent seizures after surgery despite complete removal of all lesions^9^. It is possible that a wider area beyond the main mass and its SLs, such as the cortical gyrus or central lobe containing the tumor, may be primed to develop seizures as well as tumors.

Recent genomic studies indicate that pediatric LGGs, including LEATs, originate from a single genetic alteration in *BRAF*, *MYB*, or *FGFR1* that ultimately upregulates the RAS/MAPK pathway^10–12^. Activating mutations and intragenic duplication of *FGFR1* are observed in 58% of DNETs^13^. *FGFR1-TACC1* fusion transcripts are also described in DNETs and one study reported that alterations of *FGFR1* (mutation, intragenic duplication, or fusion) were observed in up to 82% of DNETs^11^. FGFR1 encodes a receptor tyrosine kinase and acts as an oncogenic driver in various types of human cancers^14^. Therefore, alterations of *FGFR1* can be regarded as the main event in the pathogenesis of DNETs.

Comparative lesion sequencing and phylogenetic analysis have emerged as robust tools for inferring the pathogenesis and evolution of a tumor^15,16^. These approaches have been successfully applied to disclose clonal evolution from primary to metastatic tumors or from primary to recurrent malignant tumors such as lung cancer^17^ and glioblastoma^18^. However, this inference has not yet been applied to pediatric LGGs, including LEATs, because the multifocal primary development of LGGs is a rare phenomenon, and the low mutational load of LGGs can undermine the phylogenetic analyses.

To elucidate the pathogenesis of DNET and its SLs, we obtained separate biopsies from the main masses and SLs of 3 patients with DNETs. Two of the patients had tumor recurrence, and spatiotemporal profiling of mutational events in the main masses and SLs was performed. Next-generation sequencing revealed that the main masses and all SLs shared the same *FGFR1* mutations or the same intragenic duplication. Phylogenetic analyses indicated that the main masses and SLs were separated early in the pathogenesis, supporting multifocal development of DNET nodules from a single oncogenic mutation in cerebral organogenesis. These findings suggest that multifocal development may be a pathogenetic mechanism underlying the development of DNET and its satellite lesions.

## Methods

### Patient specimens

Patient specimens (tissues and matched blood) and clinical-pathologic data were retrieved from the Brain Bank of Seoul National University Hospital (SNUH) from 1997 to 2020. Written informed consent was obtained from all patients enrolled in Brain Bank of SNUH. The research protocol of this study was reviewed and approved by the institutional review board of the SNUH (IRB No. 2106-040-122) and performed in accordance with the Declaration of Helsinki. All methods were carried out in accordance with relevant guidelines and regulations. This study included 3 patients diagnosed with DNET. Thin-section, three-planar 3.0-T brain MRI was taken before surgery to delineate the tumor margin and SLs. All patients had a tumor involving the brain cortex with one or several SLs. During tumor resection, different regions of the main mass were separately biopsied. SLs were removed if possible and biopsied separately. The degree of resection was assessed by postoperative MRI. Informed consent was obtained from the legal guardian of each patient for tumor tissue extraction and analyses. Routine pathological examination was performed on the tissues from the main masses and SLs. DNA sequencing and RNA sequencing were also performed on each tissue sample. The SLs were usually very small, so all the analyses could not be done for all tissue samples. The sample designation and studies for each sample are summarized in Suppl. Table 1.

### DNA sequencing data analysis

Sequenced reads were aligned to the GRCh37 reference through the BWA-MEM algorithm (v. 0.7.15)^19^. Aligned reads were sorted and indexed with SAMtools (v. 1.6)^20^. To reduce PCR bias, duplicate reads were marked by Picard (v. 2.1.1)^21^. The reads followed the GATK (v. 3.8.0)^22^ workflow for the following analysis.

### Identification of SNVs and indels

Somatic and germline variants were detected by MuTect2 and HaplotypeCaller provided by GATK, respectively. After calling the variants, SNVs and indels were annotated with cosmic 86^23^, ExAC^24^ and gnomAD^25^ by ANNOVAR^26^. To diminish false positive variants, both somatic and germline mutations were filtered with the following conditions: (1) the variants should be located in exonic or splicing regions, (2) the population allele frequency should be less than 0.01 in ExAC EAS, gnomAD EAS, and the Korean population, and^27^, and (3) somatic variants should pass the MuTect filter conditions.

### Copy number variants analysis

Copy number variants (CNVs) were detected by both CNVkit^28^ and CoNIFER^29,30^ to verify the CNVs more accurately. RPKM was calculated with CoNIFER. RPKM was normalized into zRPKM, and then transformed into a log2 ratio to find all the valid CNVs. CNVkit was used with the default setting, and the circular binary segmentation method was selected to inter the CNVs at the segment level.

### RNA sequencing data analysis

The transcriptome was aligned to the GRCh37 reference with STAR aligner (v. 2.6.0a)^31^. Expression was quantified with the RSEM package (v. 1.3.1)^32^. RNA fusion was detected with STAR-Fusion (v. 1.4.0)^33^. To verify the significant fusions, they were filtered with several conditions: (1) one of the fusion genes had to be a protein coding gene, (2) fusions were excluded if they had a 0 read count for spanning reads, and (3) if the fusion had the same read counts at the same position, it was considered a false positive fusion. Samples of dysplastic but non-tumorous brain tissue from 4 patients were designated as controls (Suppl. Table 2).

### Phylogenetic tree analysis

The PHYLIP computational phylogenetics package was used to infer phylogenies of multiple tumor samples^34^. The maximum parsimony method was employed to construct the phylogeny of multiple tumors of a patient depending on the presence or absence of mutations. The Jaccard similarity index (JSI) method was used for gauging the differences and similarities between multiple tumors.

## Results

### Patient profile and pathological features of SLs

**Figure 1:**
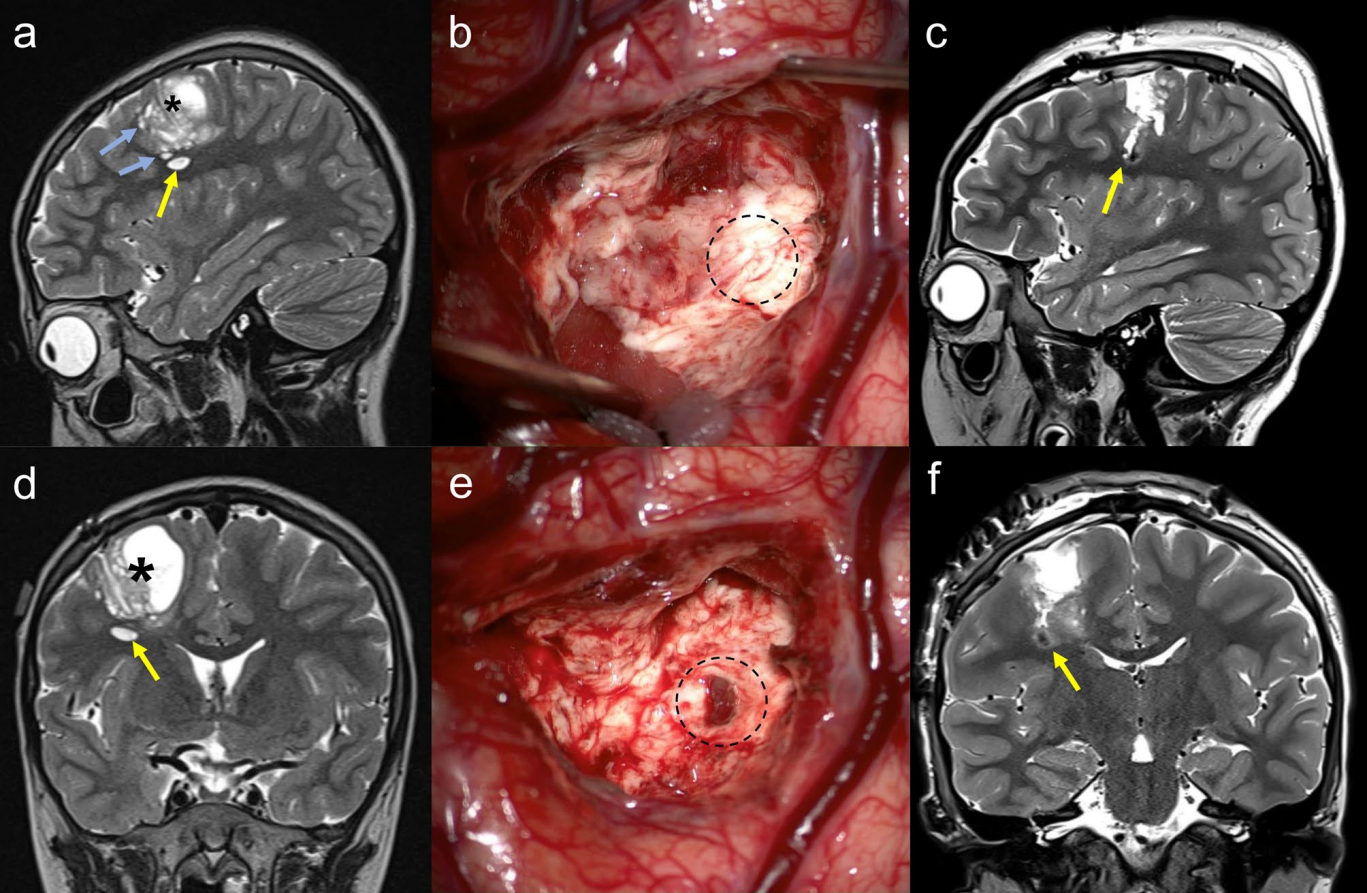
Preoperative MR images of Case D01 (**a** sagittal, **d** coronal image). A large solid and cystic mass (asterisk) involved the motor cortex and adjacent white matter. A discrete satellite lesion was located beneath the main mass (yellow arrow). There were numerous other small satellite lesions (light blue arrows). (**b**, **e**) Intraoperative photos. (**b**) After removing the majority of the main mass, a normal-looking layer of white matter was observed on the lateral-inferior boundary (dotted circle). e Trespassing the white matter layer, a dark-colored tumor was found, corresponding to the satellite lesion in (**a**) and (**b**). Postoperative MR images (**c** sagittal, **f** coronal) show the disappearance of the satellite lesion. The space is filled with the dark hemorrhagic signal.

Four patients with a LEAT and image-documented SLs were included. All patients were under 15 years of age and presented with focal-onset seizures as the chief complaint (Suppl. Table 2). All tumors were located in the cerebral cortex, and one large tumor occupied the temporo-insular cortex (D03). All patients had more than one SL separated from the main tumor. During surgery, large SLs were searched for across the intervening white matter and were removed (Fig. 1). Intraoperative ultrasonography was helpful for guidance. Not all SLs were removed because of the risk of neurological deficits or an inability to locate them intraoperatively. The degree of surgical resection was determined according to the remnant mass and/or number of SLs remaining on postoperative MRI.

**Figure 2:**
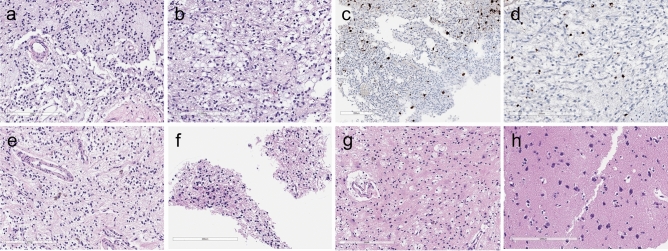
Pathological features of DNET (D01) at the first and second operations. The patient underwent two operations due to tumor recurrence. (**a**) and (**b**) The main tumor (M1) and a satellite nodule (SL1) at the first operation show the same histology composed of oligodendrocyte-like cells (OLCs) in the myxoid background. (**c**) and (**d**) NeuN-positive floating neurons are small and are hardly seen in DNET (**a**, **c** M1; **b**, **d** SL1). In the recurrent tumor, (**e**) the main tumor (rM3) and (**f**) a large SL (rSL1) show the same appearance as typical DNETs, which are composed of monotonous OLCs with floating neurons. The other, smaller SLs, (**g**) rSL3 and (**h**) rSL5 are not tumors, but contain reactive gliosis and foamy macrophage infiltration. (**a**, **b**, **e**–**h** H & E stain; **c**, **d** NeuN immunohistochemistry; Scale bar: 200 μm).

**Figure 3:**
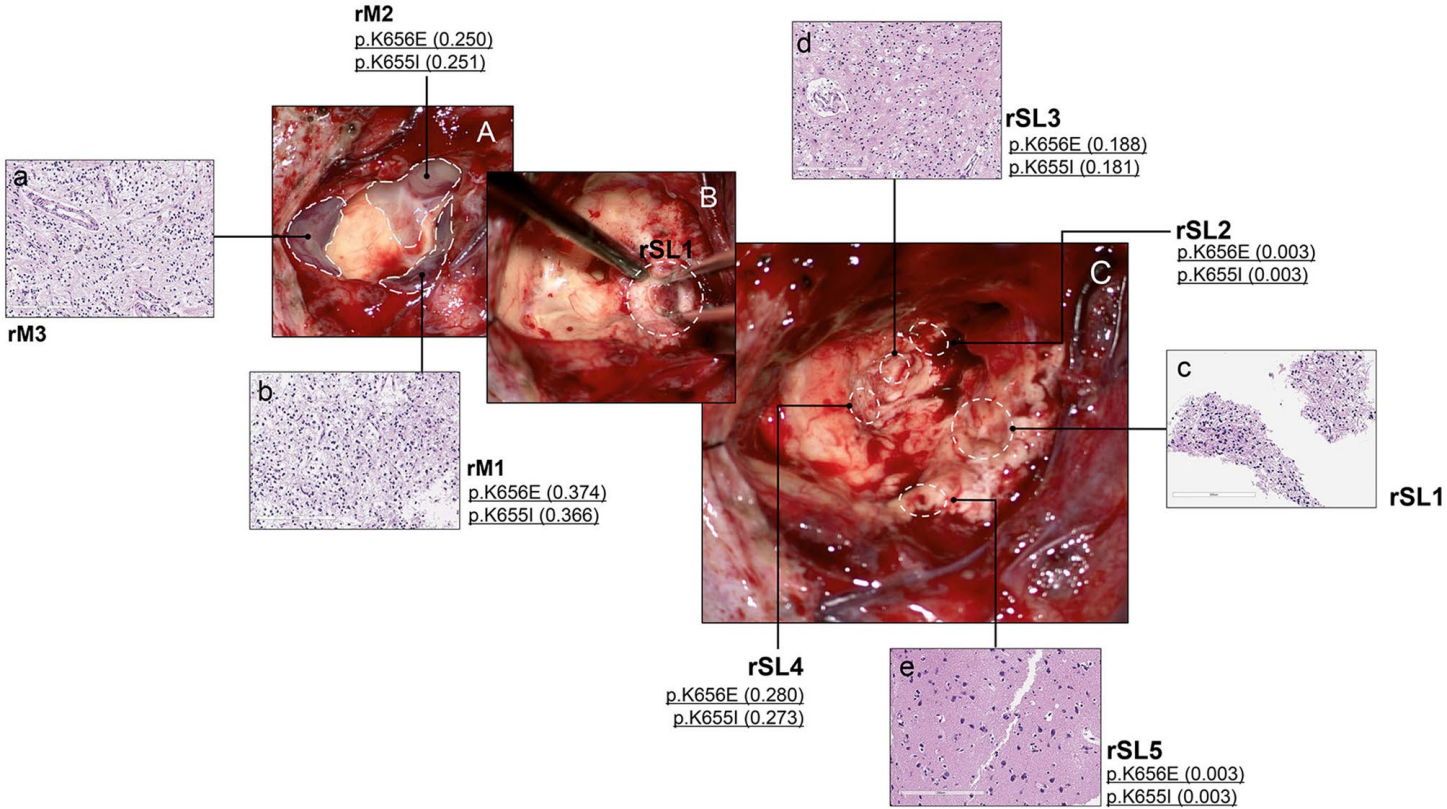
Intraoperative photographs and histological/genetic exams of Case D01 at the second operation. The patient developed multifocal recurrence in the previous tumor cavity along the resection margin (A). The main tumor was divided into 3 parts (lateral (rM1), anterior (rM2), and medial (rM3)). After removal of the main masses, hidden satellite lesions (SLs) were further searched for and removed as much as possible. SL1 was most conspicuous having formed a discrete mass (B) and the others (SL2-5) were smaller, consisting of tiny nodules. The locations of SLs are marked by dotted circles after the completion of tumor resection (C). If a tumor sample was sequenced, the presence of *FGFR1* mutation and VAF is described. Both medial and lateral masses show the same histology composed of OLCs in the myxoid background with the small number of floating neurons, consistent with DNET (**a**, **b**). SL1 has similar tumor histology, filled with OLCs and floating neurons (**c**). WES revealed mutations in the *FGFR1* gene. SL2 and SL4 were too small, and only WES was performed without histologic examinations, which revealed the same *FGFR1* mutations as SL1 had. In SL3 and SL5, histologic examinations reveal only gliosis and foamy macrophage infiltration without obvious DNET-like features, but *FGFR1* mutations are also found in SL3 and SL5 (**d**, **e**).

Three patients were histologically diagnosed with DNET. D01 was most extensively biopsied during the first and second operations. In addition to multiple portions of the initial and recurrent tumors, we obtained tissues from 7 SLs (2 from the first and 5 from the second operation). Pathological examination was performed on 4 SLs (1 from the first operation (SL1) and 3 from the second operation (rSL1, rSL3, rSL5). Upon the first operation, the main tumor (M1) and SL1 showed the same histology, composed of specific glioneuronal elements. Floating neurons were rarely observed (Fig. 2). Figure 3 depicts the specific locations of each tumor sample of D01 during the second operation. The main tumors (rM1 and rM3) had the same histology as the initial tumor (M1). Of the 3 SL samples biopsied in the recurrent tumor, rSL1 showed monotonous oligodendrocyte-like cells (OLCs) and floating neurons, which are typical features of DNET, but rSL3 and rSL5 exhibited only reactive gliosis and foamy macrophage infiltration. rSL1 (with typical DNET histology) was larger than other SLs and had a dark color, whereas rSL3 and rSL5 were tiny lesions with a pale, light gray color.

In D02, the main tumor and one SL were histologically examined. Typical features of DNET were found in both the main tumor and SL. In D03, WES and RNA-seq were applied to the initial and recurrent main tumors and a SL, but no pathological examination was done on the SL.

### Comprehensive genomic profiling of DNETs

**Figure 4:**
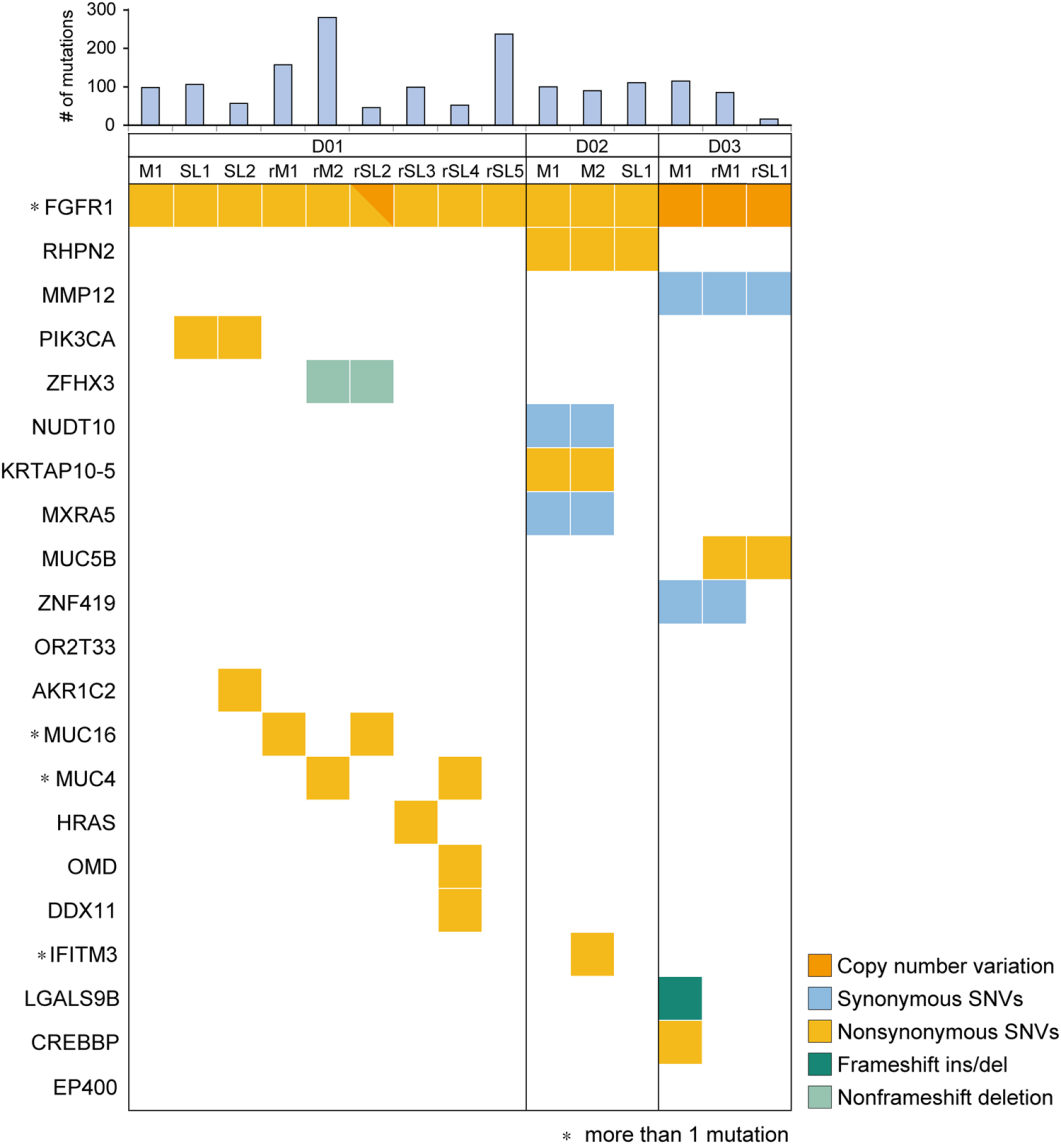
Genetic alterations in DNET, including their satellite lesions. *FGFR1* mutation was found in all DNET samples (main masses and SLs) of D01 and D02. The same *FGFR1* duplication was detected in the main mass and SL of D3.

FGFR1 alterations were identified in all three DNET patients. FGFR1 K656E and K655I mutations were harbored as compound heterozygous in cis in D01 and D02. We inspected whether compound heterogeneity affected expression, but both alleles were expressed equally (Suppl. Table 3). All sequenced SLs in D01 (SL1, SL2, rSL2, rSL3, rSL4, and rSL5) and D02 (SL) had the same mutations as their mother tumors (Fig. 4). For the D01-rSL5 sample, FGFR1 mutations were not found with a mutation caller. However, a read that contained compound heterogeneity was confirmed manually (Suppl. Table 3). In D03, FGFR1 mutations were not detected but a copy number alteration was discovered; amplification of FGFR1 was confirmed in all three samples of D03: M1, rM1, and rSL1 (Suppl. Figure 1). Copy number gain was partially detected in FGFR1 including the tyrosine kinase domain. To find the genes with driver mutations in DNET, we selected the genes that are reported in the COSMIC database, especially from among the census gene list or central nervous system tumor-related genes. The selected genes were in Oncomap, and among them only FGFR1 had alterations that were shared by all DNET patients (Suppl. Table 4 and Suppl. Figure 2).

### Distinct molecular features of multiple tumors in each patient

Some SLs were too small for us to process RNA sequencing for all samples; we obtained at least two samples from each patient (Suppl. Table 1). The expression patterns were completely divided into groups by the first two principal components (Suppl. Figure 3). PC1 accounted for 42% and PC2 accounted for 24% of the variation (Suppl. Figure 4). All tumor components, whether they were from the main mass or SL, clustered into each tumor entity, apparently separated from the 4 control brain samples. This indicated that the SLs contained true tumor tissues different from the dysplastic brain.

### Each tumor separated from the initial stage of tumor progression

**Figure 5:**
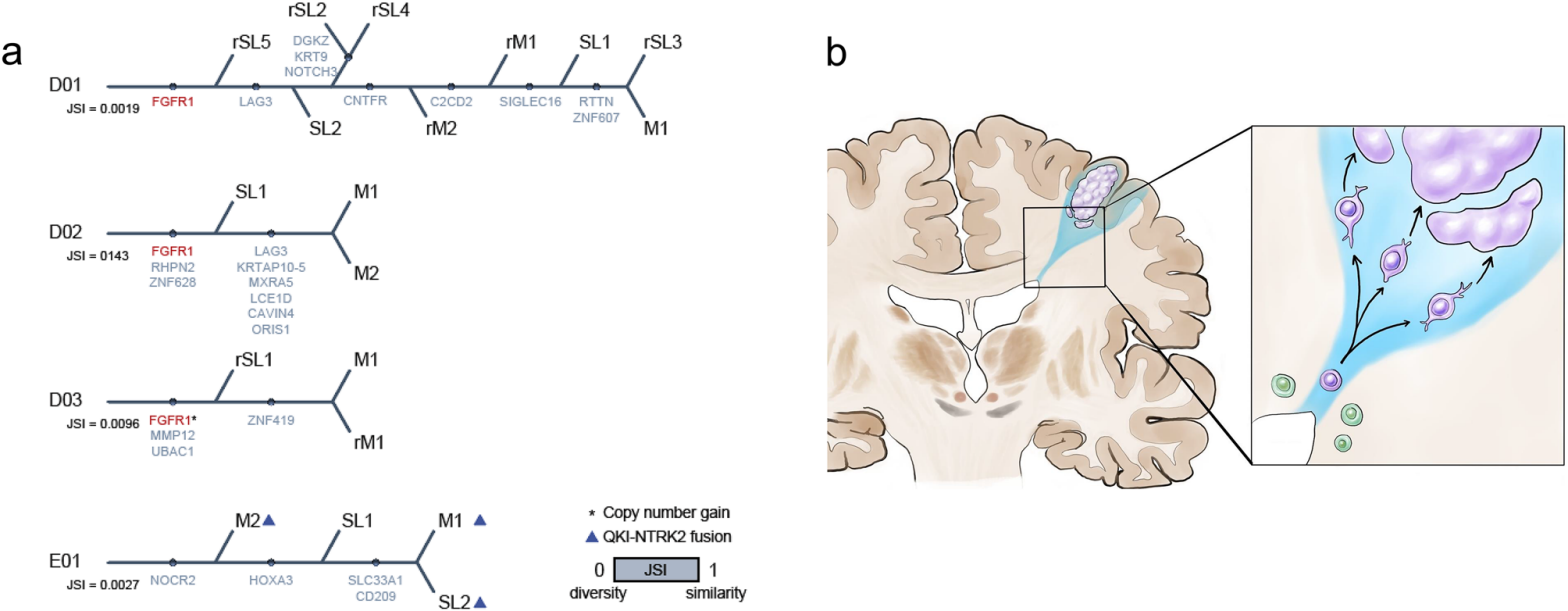
(**a**) The trees were constructed manually according to the number of shared mutations. Jaccard’s similarity index was computed from the total number of mutations and shared mutations. The red text marks the known driver gene. (**b**) The theoretical model for the tumor progression of DNET. Dividing stem/progenitor cells share the same oncogenic alteration(s) and then migrate to cortical layers, and later undergo tumorigenesis in multiple foci independently.

All tumor samples shared the same genomic alteration of *FGFR1* in DNET, regardless of the space (main tumor or SL) and time (primary or recurrence). To reveal the pathogenesis of the multiple lesions in each patient, we attempted to estimate the genetic distances of the tumors with mutations. Due to the low mutation frequency and small number of shared mutations, it was hard to examine the clonal evolution with statistical inference methods, so we drew the tree manually (Fig. 5a). We expected that SLs or recurrent tumors would be shown to have stemmed from the primary main tumor; however, the trees showed that multiple tumors had no significant relationship with either space (main mass/SL) or time (primary/ recurrent). We further validated the similarity and diversity of the multiple tumors by calculating the Jaccard similarity index (JSI). The JSI was close to 0, which meant that the tumors were completely disparate from each other, the value of JSI of D01, D02 and D03 is 0.0019, 0.0096 and 0.0143, respectively. These results supported the hypothesis that the main mass and its SLs separated at an early stage in oncogenesis, with shared *FGFR1* alterations, and then they further developed independently in different places (Fig. 5b).

## Discussion

In 1996, Barkovich et al. proposed a comprehensive scheme for the classification of malformations in human cortical development^35^. Interestingly, LEATs (DNETs and gangliogliomas) were included in the scheme as malformations due to abnormal cell proliferation because these tumors are frequently accompanied by true cortical dysplasia adjacent to the tumor mass. This composite architecture of DNET was well illustrated in the original description by Daumas-Duport^4^. She depicted DNET as a tumor core (so-called specific glioneuronal element) surrounded by a rim of cortical dysplasia with small nodular foci at the bottom (inner side) of the mass. A later study in the MRI-era noticed the presence of smaller nodules in the vicinity of the tumor but separate from it^36^. Bonn’s group emphasized that small pseudocystic nodules are located in the neighboring cortex or subcortical white matter in nearly 60% of DNETs^37^. Although the presence of separated nodules around DNETs has been recognized for decades, the clinical meaning or histopathological features of these SLs have not been seriously studied before. Daumas-Duport described that the inner nodular foci of DNETs were devoid of specific glioneuronal elements but were composed of astrocytes and oligodendrocytes, sometimes indistinguishable from pilocytic astrocytomas^4^. However, it is not clear whether the so-called glial nodules of composite DNETs that Daumas-Duport described were the same nodules that many authors had observed on MRI, obviously separated from the tumor. Recently, we discovered the clinical significance of SLs of DNETs^9^. The higher recurrence rate when the SLs were not removed indicated that the SLs were also tumor tissues. Some tumors directly recurred from the remnant SLs, but there was no growth of remnant SLs in other patients, implying that the histologic nature and tumorigenic potentials of SLs are diverse as demonstrated in Figs. 2 and 3.

Hotspot mutations of *FGFR1* (K656E, K655I, and N546K) were initially identified in the germline DNA of rare familial DNET patients and were further identified in somatic tumor archives^13^. Approximately 58% of DNETs in the archival registry had alterations in *FGFR1* (point mutations, duplications, and gene fusions), while BRAF alterations were absent in DNETs. This result was in striking contrast with previous studies that 30% of DNETs had the *BRAF* V600E mutation or copy number gain^38,39^. Qaddoumi et al.^11^ found FGFR1 alterations in 82% of pathologically diagnosed DNETs and that BRAF alterations were uncommon in DNETs (9%). Stone et al.^40^ reported that *FGFR1* mutations were found in 75% of DNETs. The different proportions of *FGFR1* and *BRAF* alterations in previous studies reflect the obscurity in the pathological diagnosis of DNET. Differential diagnosis of LEATs, including DNET, has notoriously poor interobserver agreement^41^. Pediatric LGGs harboring OLCs are a very heterogeneous group of tumors. Pediatric oligodendroglioma typically lacks *IDH*1/2 mutations and 1p/19q co-deletion, making a differential diagnosis from DNET precarious^42,43^. Therefore, current genomic studies suggest that FGFR1-alterations seem to be the single defining genetic event in DNET pathogenesis.

In our study, SLs showed heterogeneous histology from dysplastic cortex to true DNET. However, all SLs examined had the same *FGFR1* mutation as the mother tumor. Variant allele frequencies (VAFs) of *FGFR1* mutations were variable with a trend toward lower VAF values in SLs with rather normal-looking histology. This indicates that the proportion of cells with pathogenic mutations may be important to the histologic alterations and oncogenesis. Clinically, some SLs do not grow for years on imaging follow-up, while others enlarge steadily, reflecting diverse proliferation potentials. Otherwise, SLs may result from daughter nodule formation connected to the mother tumor, at least microscopically. Resection of the whole gyrus and thorough histologic examination may solve the problem, but this may not be feasible because many SLs are located on the medial side of the tumor, often deep in functionally important white matter tracts.

Because all tumors and SLs share the same *FGFR1* mutations in each patient, we can assume that *FGFR1* mutation is one of the earliest events in the development of DNET and its SLs. Our analyses showed that SLs develop independently from the main mass portions. Nevertheless, we considered that the multifocal development hypothesis more likely because SL and mother tumor barely shared the mutation except FGFR1 mutation and the mutated genes types were completely different from each other. Although the cell of origin for DNET is unknown, we can assume that a neural or neuroglial progenitor cell with *FGFR1* alterations and its daughter cells moved to a cortical area and were dormant for several years until multifocal tumors develop. Alternatively, the dispersed daughter cells make several tumor nodules with very low growth potential that become clinically significant years later. We and others observed no additional genetic driver mutations in DNETs and the growth rate of DNET is known to be very slow, supporting the second scenario.

While our analyses indicate that multifocal development is the most likely mechanism underlying the formation of SLs in our study population, this does not exclude the possibility that a mechanism of infiltration/extension, either independently or in tandem with multifocal development, may still occur in the DNET population at large. While the overall rarity of this tumor makes it challenging to obtain larger sample sizes, additional and/or larger studies may help support the predominance of the mechanism we propose. Future studies may also consider sampling intervening white matter tracts between the mother lesion and SLs show that these tracts do not contain evidence of occult extension. This would more definitively disprove a theory of infiltration/extension. However, the ability to perform this approach would be contingent upon confirmation that intervening tracts do not represent areas of the eloquent cortex, which are not amenable to surgical resection.

In conclusion, DNETs have a composite structure of a main mass and surrounding SLs. Separate sampling and sequencing showed that alterations in driver oncogenes such as FGFR1 were shared by both the main mass and its SLs. Phylogenetic analyses indicated that the main mass and SLs develop rather independently, arguing against the infiltration/ extension model. Our results indicate that progenitor cells with a priming genomic alteration migrate to and proliferate in the brain cortex and later develop into glioneuronal tumors with SLs.

## Supplementary Information

Supplementary Information 1.

Supplementary Information 2.

Supplementary Information 3.

Supplementary Information 4.

Supplementary Information 5.

Supplementary Information 6.

Supplementary Information 7.

Supplementary Information 8.

Supplementary Information 9.

## Data Availability

Generated WES data have been deposited in the Sequence Read Archive (SRA) data under the accession number PRJNA860303, which will be released after publication (https://dataview.ncbi.nlm.nih.gov/object/PRJNA860303?reviewer=3lcvinfngujjldii2f7gdgf7co). All the data that supports results and conclusions for this study are included in this article and supplementary information.
