# Author Correction: Genomic analysis as a tool to infer disparate phylogenetic origins of dysembryoplastic neuroepithelial tumors and their satellite lesions

**DOI:** 10.1038/s41598-023-28950-0

**Published:** 2023-01-31

**Authors:** Yeajina Lee, Jeyul Yang, Seung Ah Choi, Seung‐Ki Kim, Sung-Hye Park, Hyun Joo Park, Jong-Il Kim, Ji Hoon Phi

**Affiliations:** 1 Department of Biomedical Sciences, Seoul National University Graduate School, Seoul, Republic of Korea; 2 Genomic Medicine Institute, Medical Research Center, Seoul National University, Seoul, Republic of Korea; 3 Neuro-Oncology Clinic, National Cancer Center, Goyang, Republic of Korea; 4 Division of Pediatric Neurosurgery, Seoul National University Children’s Hospital, Seoul National University College of Medicine, Seoul, Republic of Korea; 5 Department of Neurosurgery, Seoul National University College of Medicine, Seoul, Republic of Korea; 6 Neuroscience Research Institute, Medical Research Center, Seoul National University College of Medicine, Seoul, Republic of Korea; 7 Department of Pathology, Seoul National University College of Medicine, Seoul, Republic of Korea; 8 Department of Biochemistry and Molecular Biology, Seoul National University College of Medicine, Seoul, Republic of Korea

Correction to: Scientific Reports, 10.1038/s41598-022-26636-7, published on 13 January 2023

In the original version of this Article author Yeajina Lee was omitted as an equally contributing author.

“These authors contributed equally: Jeyul Yang and Seung Ah Choi”

now reads:

“These authors contributed equally: Yeajina Lee, Jeyul Yang and Seung Ah Choi”

The original Article has been corrected.

