# Supplementary material for: Genomic analysis as a tool to infer disparate phylogenetic origins of dysembryoplastic neuroepithelial tumors and their satellite lesions
Source: Sci Rep. 2023 Jan 13;13:682. doi: 10.1038/s41598-022-26636-7 (PMC9839671; doi:10.1038/s41598-022-26636-7)
Supplement: Supplementary file 2 — Supplementary Information 2. [file 41598_2022_26636_MOESM2_ESM.docx]

| Case | Age (years) | Sex | Initial symptom | Tissue characteristics | | | Major genomic changes of tumor |
| --- | --- | --- | --- | --- | --- | --- | --- |
|  |  |  |  | Pathology | Location | Underlying pathology |  |
| D01 | 7 | M | Seizure | DNET | Frontal lobe | NA | FGFR1 K656E/K655I |
| D02 | 9 | M | Seizure | DNET | Temporal lobe | NA | FGFR1 K656E/K655I |
| D03 | 7 | F | Seizure | DNET | Temporo-insular lobe | NA | FGFR1 TKD duplication |
| Control1 | 3 | M | Seizure | Neocortex* | Temporal lobe | Ganglioglioma | NA |
| Control2 | 1 | M | Seizure | Neocortex* | Temporal lobe | Cavernous malformation | NA |
| Control3 | 5 | F | Hemiparesis | Neocortex* | Temporal lobe | Pilocytic astrocytoma | NA |
| Control4 | 9 | M | Diplopia | Neocortex* | Frontal lobe | Supratentorial ependymoma | NA |
| *Neocortex of control cases was resected for the approach to the deep-seated underlying pathology (tumor or cavernous malformation)  NA: Not applicable | | | | | | | |

Supplementary Table 2. Patient characteristics and major genomic changes of the tumors
