# Supplementary figures and images for: Genomic analysis as a tool to infer disparate phylogenetic origins of dysembryoplastic neuroepithelial tumors and their satellite lesions
Source: Sci Rep. 2023 Jan 13;13:682. doi: 10.1038/s41598-022-26636-7 (PMC9839671; doi:10.1038/s41598-022-26636-7)

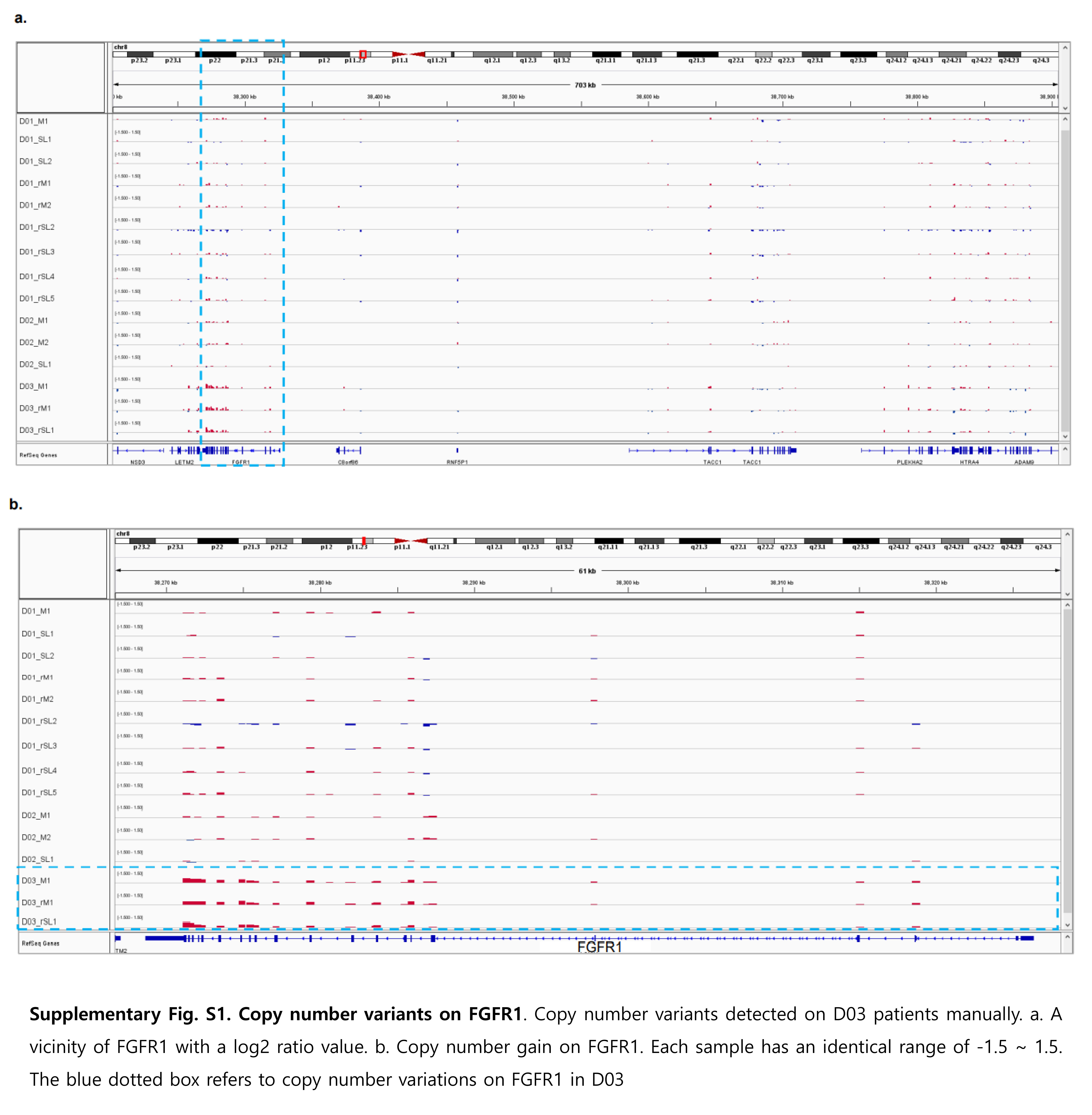

Supplement: Supplementary file 6 — Supplementary Information 6. [file 41598_2022_26636_MOESM6_ESM.jpg]

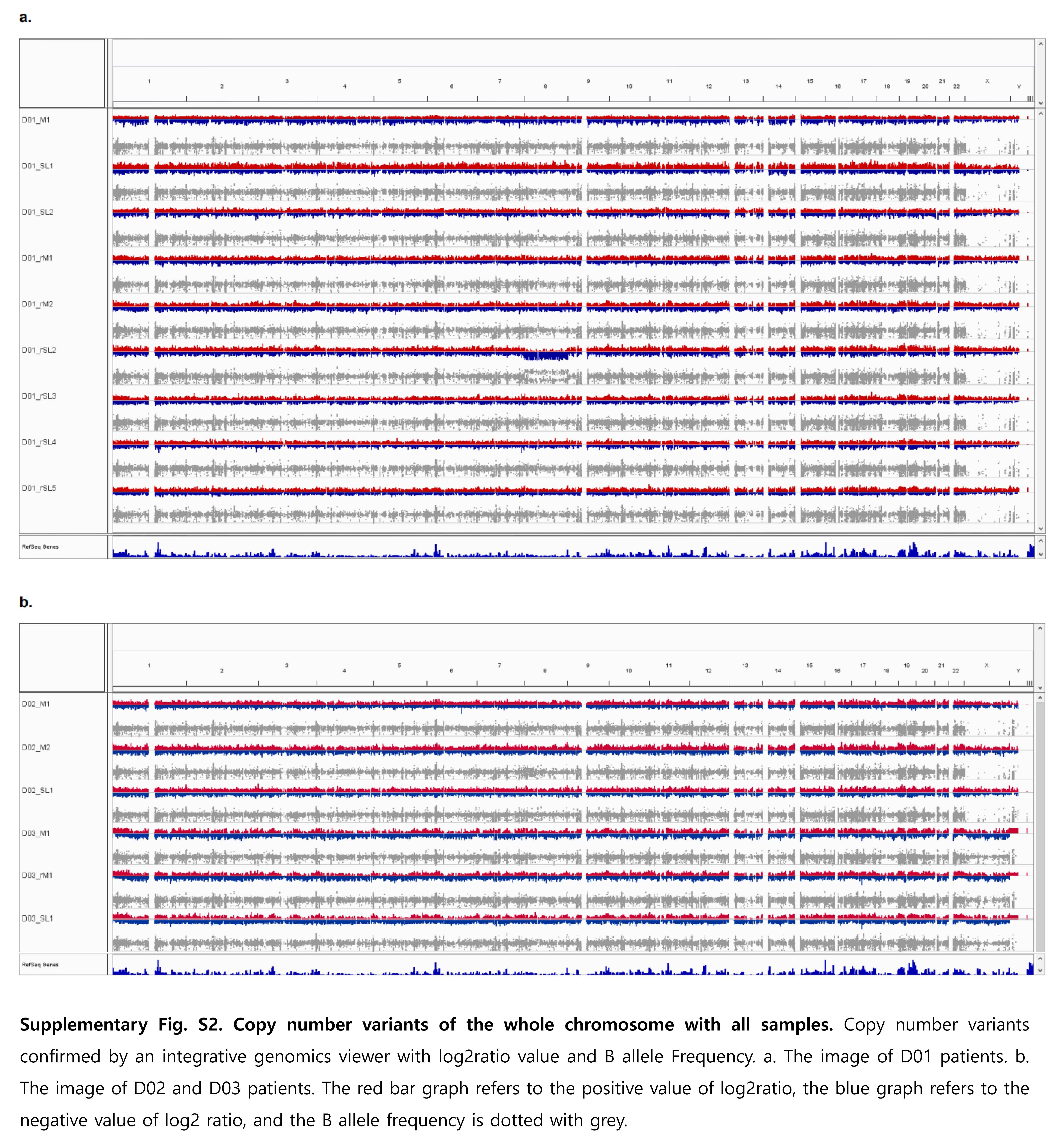

Supplement: Supplementary file 7 — Supplementary Information 7. [file 41598_2022_26636_MOESM7_ESM.jpg]

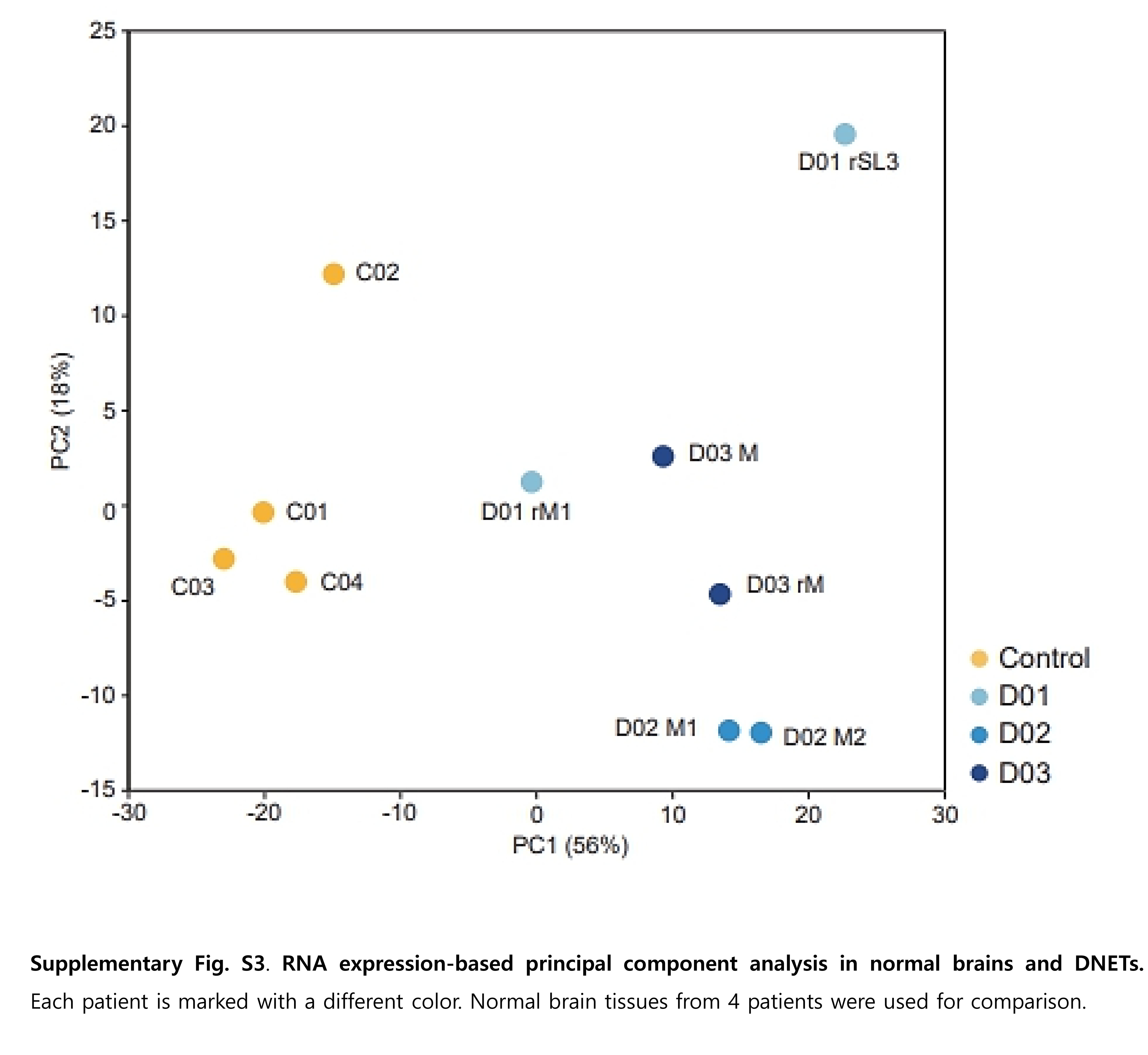

Supplement: Supplementary file 8 — Supplementary Information 8. [file 41598_2022_26636_MOESM8_ESM.jpg]

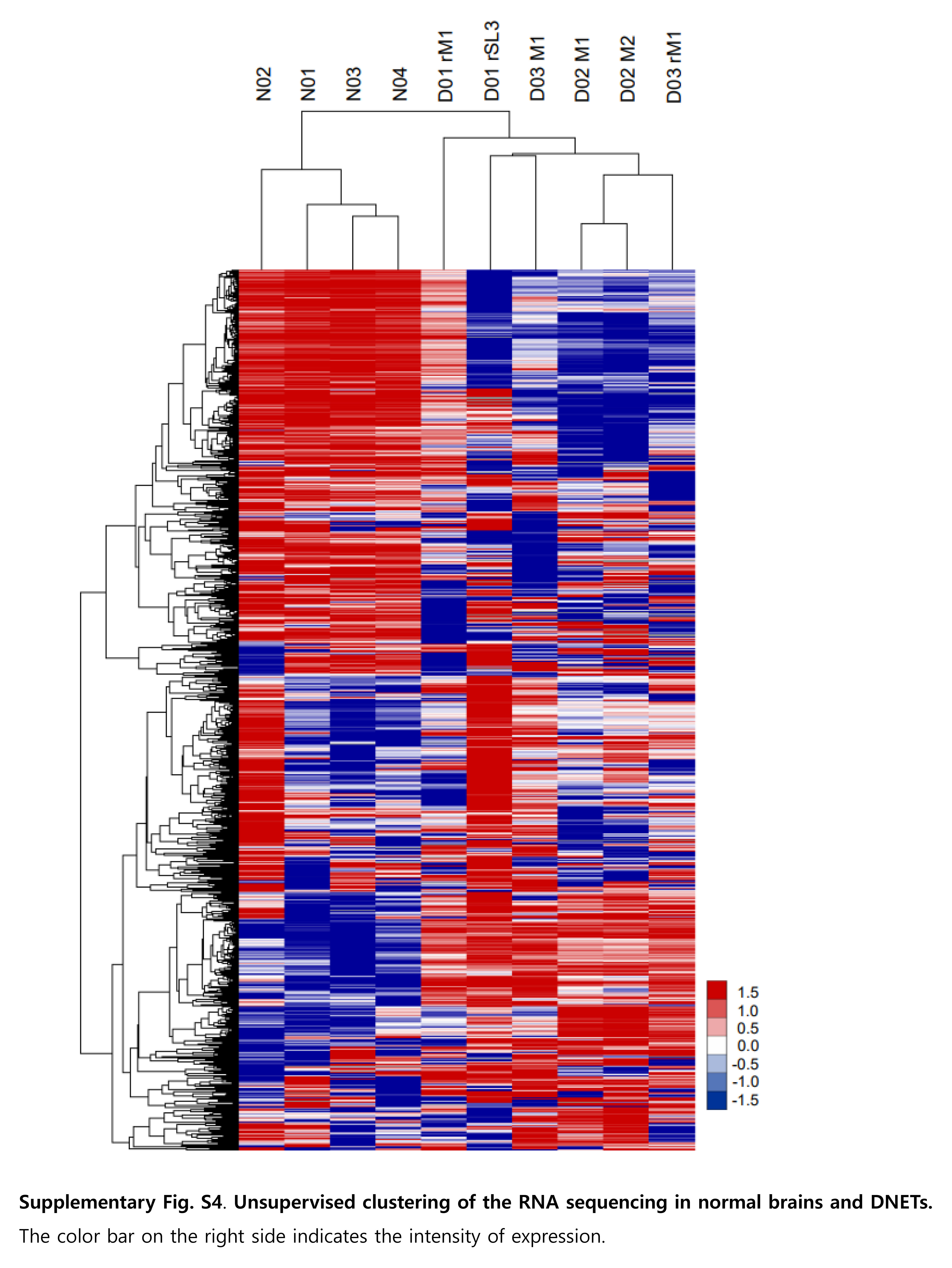

Supplement: Supplementary file 9 — Supplementary Information 9. [file 41598_2022_26636_MOESM9_ESM.jpg]
